# Protective Effects of Therapeutic Neutrophil Depletion and Myeloperoxidase Inhibition on Left Ventricular Function and Remodeling in Myocardial Infarction

**DOI:** 10.3390/antiox12010033

**Published:** 2022-12-24

**Authors:** Henning Guthoff, Alexander Hof, Anna Klinke, Martina Maaß, Jürgen Konradi, Dennis Mehrkens, Simon Geißen, Felix S. Nettersheim, Simon Braumann, Erik Michaelsson, Richard J. Nies, Samuel Lee, Marie-Christin Redzinski, Vera B. M. Peters, Harshal N. Nemade, Philipp von Stein, Holger Winkels, Volker Rudolph, Stephan Baldus, Matti Adam, Martin Mollenhauer

**Affiliations:** 1 Department for Experimental Cardiology, Faculty of Medicine, University of Cologne, and Clinic III for Internal Medicine, University Hospital Cologne, 50937 Cologne, Germany; 2 Center for Molecular Medicine Cologne (CMMC), Faculty of Medicine and Faculty of Mathematics and Natural Sciences, University of Cologne, 50931 Cologne, Germany; 3 Clinic for General and Interventional Cardiology/Angiology, Agnes Wittenborg Institute for Translational Cardiovascular Research, Herz- und Diabeteszentrum NRW, University Hospital of the Ruhr-Universität Bochum, 32545 Bad Oeynhausen, Germany; 4 Division of Dry-Eye and Ocular GVHD, Department of Ophthalmology, Faculty of Medicine and University Hospital Cologne, University of Cologne, 50937 Cologne, Germany; 5 Early Clinical Development, Research and Early Development, Cardiovascular, Renal and Metabolism (CVRM), BioPharmaceuticals R&D, AstraZeneca, Z4-46798 Gothenburg, Sweden

**Keywords:** myocardial infarction, ischemic cardiomyopathy, myocardial remodeling, polymorphonuclear neutrophils, myeloperoxidase, MPO inhibition, AZM198

## Abstract

Myocardial infarction (MI) is a leading cause of morbidity and mortality worldwide. Improved survival has led to an increasing incidence of ischemic cardiomyopathy, making it a major reason for hospitalization in the western world. The inflammatory response in the ischemic myocardium determines the extent of structural remodeling and functional deterioration, with neutrophils (PMN) being a key modulator of the propagation and resolution of inflammation. The heme enzyme myeloperoxidase (MPO) is abundantly expressed in PMN and is an important mediator of their inflammatory capacities. Here, we examine the effects of PMN reduction, MPO deficiency and MPO inhibition in two murine models of MI. Reduction in PMN count resulted in less scar formation and improved cardiac function. Similar results were obtained in genetically MPO deficient mice, suggesting that MPO is a critical factor in PMN-mediated cardiac remodeling. To test our findings in a therapeutic approach, we orally administered the MPO inhibitor AZM198 in the context of MI and could demonstrate improved cardiac function and reduced structural remodeling. Therefore, MPO appears to be a favorable pharmacological target for the prevention of long-term morbidity after MI.

## 1. Introduction

Myocardial infarction (MI) is a leading cause of morbidity and mortality worldwide, mainly due to coronary artery disease and myocardial ischemia [1]. Novel interventional reperfusion therapies have significantly improved survival [2]. Nonetheless, as overall life expectancy has greatly increased over recent decades, the incidence of ischemic cardiomyopathy and heart failure (HF) has grown, affecting ≥64 million individuals worldwide [3]. Today, HF is a major reason for hospitalization among adults and the elderly in western countries [4].

Developing HF after ischemic injury is due to a complex interplay between myocardial inflammation, degradation and remodeling [5]. Acute loss of cardiomyocytes after MI leads to pro-inflammatory cytokine release from necrotic cells, activating innate immune pathways and stimulating an extensive inflammatory response [6]. Among the first cells to accumulate in the ischemic myocardium are polymorphonuclear neutrophils (PMN) [7]. PMN subset composition has been proven to be essential for a balanced inflammatory and reparative immune response after myocardial ischemia [8,9]. They secrete a variety of inflammatory mediators and oxidative enzymes, such as myeloperoxidase (MPO), thereby generating high levels of reactive oxygen species (ROS), which can further promote myocardial injury. In addition, they attract other innate immune cells, e.g., monocytes to the ischemic area, and thus activate reparative pathways and ensure phagocytotic removal of necrotic cell debris [8]. PMN are essential for the initiation of myocardial repair processes after ischemic injury and their full depletion by Ly6G antibody treatment boosts adverse ventricular remodeling and scar formation [10]. In contrast, by fine modulation of inflammatory capacities, PMN can ameliorate cardiac deterioration in a model of permanent coronary vessel occlusion [11].

MPO is a heme enzyme abundantly expressed in and released by PMN and monocytes and is a key mediator of inflammation. In the presence of its principal substrate, hydrogen peroxide (H_2_O_2_), MPO catalyzes the production of ROS such as hypochlorous acid (HOCl) and thereby exhibits cytotoxic properties [12]. Furthermore, MPO plays a critical role in the modulation of vascular function by limiting the bioavailability of nitric oxide (NO), an important anti-inflammatory and vasodilating agent, thus promoting endothelial dysfunction and cardiovascular disease [13,14]. MPO has been shown to play a pivotal role in leukocyte recruitment and maladaptive structural remodeling of the infarcted myocardium [15]. Its catalytic function has been linked to the activation of proteolytic enzymes such as matrix-metalloproteinases [16,17] and fibroblast-to-myofibroblast transdifferentiation [18] and the promotion of ventricular arrhythmias after MI [18]. Due to the various pathophysiological effects of MPO on cardiovascular disease, pharmacological inhibition has come into scientific focus as a potential therapeutic option [19]. Thioxanthines are a class of suicide substrates that irreversibly inactivate MPO through modification of its heme groups and thereby inhibit HOCl production [20]. They have already passed phase I clinical trials and are currently being investigated in phase II studies in HF with preserved ejection fraction (NCT03756285) [21,22]. Further preclinical studies have shown beneficial effects of MPO inhibition on atherosclerotic plaque stability and pulmonary vascular function [23,24,25].

Here, we aim to evaluate the influence of PMN reduction, MPO deficiency and MPO inhibition on myocardial remodeling in two murine models of cardiac ischemic injury. A reduction in PMN count resulted in better preserved integrity of left ventricular structure and function in mice. Similar results were seen in MPO deficient (*Mpo*^−/−^) mice, suggesting that MPO is a critical factor in PMN mediated cardiac remodeling. To test our findings in a therapeutic approach, we orally administered the MPO inhibitor AZM198, a thioxanthine derivative, in the context of experimental MI, by which we could demonstrate improved cardiac function and reduced structural remodeling.

## 2. Materials and Methods

### 2.1. Animal Studies and Ethics Statement

Male, 8- to 12-week-old *Mpo*^−/−^ and wildtype (WT) mice were used for all animal studies. Animals were of C57BL/6J background (Jackson Laboratory, Bar Harbor, ME, USA). The strategy for the generation of *Mpo*^−/−^ mice has been described previously [26]. All animal studies were approved by the local authorities (State Agency for Nature, Environment and Consumer Protection (LANUV), Recklinghausen, NRW, Germany) and the University Cologne Animal Care and Use Committees. All surgical interventions were performed under anesthesia using isoflurane and perioperative analgesia with buprenorphine to minimize suffering.

### 2.2. Left Anterior Descending Artery Ligation

Mice were anesthetized with isoflurane, received low dose buprenorphine subcutaneously (Essex-Pharma, Munich, Germany; 0.05 mg/kg bodyweight) for analgesia and were placed on a heating pad to regulate body temperature. Following endotracheal intubation, animals were ventilated with 150 strokes/min and stroke volume of 7 μL/g bodyweight (Harvard Apparatus, Holliston, MA, USA). Surgical procedures were carried out using a dissecting microscope (Leica MZ6, Leica Microsystems, Wetzlar, Germany).

(1) Permanent left anterior descending artery (LAD) ligation (PI): After lateral thoracotomy of the fourth intercostal space, a suture (8/0 polypropylene suture, Polypro, CP Medical, Norcross, GA, USA) was placed around the LAD and the artery was ligated with a bow tie. Ischemia was visually confirmed by blanching of the left ventricular (LV) apex.
(2) Ischemia and reperfusion (I/R): The LAD ligation was removed after 40 min to allow up to 21 days of reperfusion.

Animals that died during instrumentations, or that did not properly recover, were excluded from analyses. Animal experiments were performed according to the Guide for the Care and Use of Laboratory Animals published by the US National Institutes of Health.

### 2.3. Ly6G Antibody Treatment

PMN reduction was performed by intraperitoneal (i.p.) injection of monoclonal anti-mouse Ly6G antibody 1A8 (250 µg; BioXcell, Lebanon, NH, USA) 2 days before LAD ligation.

### 2.4. Flow Cytometry

Blood was drawn into EDTA blood collection tubes in deep isoflurane anesthesia of mice by heart puncture. Erythrocytes were lysed by adding NH_4_Cl lysis buffer (0.83% NH_4_Cl in ddH_2_O + 0.1% KHCO_3_ + 1 mM EDTA; pH 7.4) to whole blood. Washing was performed by adding an appropriate amount of serum-free PBS (0.01 M sodium phosphate, 0.15 M sodium chloride; pH 7.2) to each sample and performing centrifugation for 10 min at 300× *g* with consecutive discarding of the supernatant. Samples were fixed with 3.7% formaldehyde solution and blocked with 10% goat serum. Primary antibody was against Ly6G (1:50, rat anti-mouse antibody, Hycult biotech, Uden, The Netherlands) and secondary antibody was goat anti-rat immunoglobulin (1:100, Alexa Fluor-488 conjugated, Invitrogen, Waltham, MA, USA). Data were acquired on a FACS Canto II (BD Biosciences, Franklin Lakes, NJ, USA), and analysis was performed with FlowJo software (Ashland, OR, USA). PMN count was analyzed relative to untreated control.

### 2.5. MPO Plasma Level

Blood was drawn in deep isoflurane anesthesia of mice by heart puncture into heparinized syringes. Plasma was analyzed for MPO using Mouse MPO ELISA (Hycult biotech, Uden, The Netherlands) according to manufacturer’s instructions.

### 2.6. Echocardiography

A commercial echocardiography system (Philips iE33 ultrasonic system, “Qlab Cardiac Analysis”-Software) equipped with a 15-MHz linear array transducer (L15-io7) was used. Mice were anesthetized with isoflurane and were placed on a heating pad to regulate body temperature. Parasternal long and short axis views of the LV in two-dimensional plane as well as M-mode were recorded. Additionally, multiple short-axis views were recorded every 500 µm for reconstructive three-dimensional echocardiography using a millimeter screw-tripod, allowing an analysis of the ejection fraction and the end systolic and end diastolic volume [27,28].

### 2.7. Pressure-Volume Loop Analyses (PV-Loop)

Mice were anesthetized with isoflurane, received low dose buprenorphine for analgesia subcutaneously and were placed on a heating pad to regulate body temperature. Following endotracheal intubation, animals were ventilated with 150 strokes/min and stroke volume of 7 μL/g bodyweight. The left jugular vein was cannulated with PE-10 tubing and a solution of 12.5% bovine serum albumin (Sigma-Aldrich Corp., St. Louis, MO, USA, 2 μL/g bodyweight) was infused. A microtip conductance pressure–volume catheter (1.4F, SPR-839 NR, Millar Instruments, Houston, TX, USA) was inserted into the carotid artery. Heart rate was maintained between 400 and 500 bpm by adjusting the concentration of isoflurane accordingly. The thorax was opened, the heart apex was punctured with a 26 G cannula and another 1.4 F microtip conductance catheter was inserted into the LV. LV pressure and volume and carotid pressure were recorded continuously with an ADInstruments PowerLab 8/30 system (ADInstruments, Spechbach, Germany). Volume calibration was performed using ADInstruments volume calibration cuvette. Cardiac output (CO) was calculated from LV pressure volume loops. Mean arterial pressure (MAP) was calculated from carotid pressure.

### 2.8. Assessment of Left Ventricular Fibrosis and Wall Thickness

Cardiac paraffin sections were stained with Masson’s trichrome following standard protocols. Images were acquired using a DP25 camera (Olympus, Hamburg, Germany) mounted on a BX51 microscope (Olympus). Mean fibrotic area was quantified using Cell A software (Olympus). Wall thickness of left ventricular myocardium was determined by measuring the thickness at 13 randomly chosen positions along the left ventricular wall in 5 heart sections per mouse, and the results were averaged. Quantification was performed in a blinded fashion.

### 2.9. Staining for Myocardial PMN Infiltration

Frozen heart sections (6 µm) in OCT compound were fixed with acetone. Sections were incubated with rat anti-mouse F4/80 (1:100, Abcam, Cambridge, UK) or with neutrophil Ly6G primary antibody (1:40, Hycult biotech, Uden, The Netherlands), and endogenous peroxidase activity was blocked. Secondary antibody was horseradish peroxidase (HRP)-labeled rabbit anti-rat (1:100, Dako, Glostrup, Denmark), and tertiary antibody was HRP-labeled goat anti-rabbit (1:500, Vectorlabs, Burlingame, CA, USA) in 3% mouse serum. PMN were stained with AEC solution and tissue was counterstained with hematoxylin. Images were acquired using a DP25 camera (Olympus, Hamburg, Germany) mounted on a BX51 microscope (Olympus). The number Ly6G positive cells was assessed in 5 heart sections per mouse and the results were averaged. Quantification was performed in a blinded fashion.

### 2.10. Immunofluorescence Staining for α-Smooth Muscle Actin, Discoidin Domain-Containing Receptor 2 and Connexin 43

Frozen heart sections (4 µm) in OCT compound were thawed, fixed with 3.7% formaldehyde solution and blocked with 10% mouse serum. Slides were treated with 0.1% Triton X-100 and incubated with either primary antibody against α-smooth muscle actin (α-SMA; 1:200, rabbit IgG, ab5694, Abcam, Cambridge, UK) and discoidin domain-containing receptor 2 (DDR-2; 1:50, goat IgG, sc7555, Santa Cruz, TX, USA) or with primary antibody against connexin 43 (Cx43; 1:1000, rabbit IgG, C6219-2 ML, Sigma-Aldrich, Burlington, MA, USA) for 1 h at room temperature in PBS with 0.1% Triton-X100 and 10% mouse serum. Secondary antibodies were Alexa Fluor-594 chicken anti-rabbit IgG and Alexa Fluor-488 chicken anti-goat IgG (Invitrogen, Waltham, MA, USA). Nuclei were stained with DAPI. Images were taken with a Retiga 1300 CCD camera mounted on Leica DMLB fluorescence microscope by iVision v4.0. Quantification of Cx43 expression was performed in 5 heart sections per mouse and the results were averaged. Quantification was performed in a blinded fashion.

### 2.11. MPO Inhibitor Treatment

MPO inhibitor AZM198 (AstraZeneca, Mölndal, Sweden) was administered ad libitum in chow at a concentration expected to yield a daily dose of 500 µmol/kg and a plasma concentration of 2 µmol/L, estimated to inhibit extracellular MPO activity by >90%. Inhibitor vs. control treatment was started at the day of MI or sham operation (d0) in 8- to 12-week-old C57BL/6J WT mice. Adequate exposure was confirmed by mass spectrometry quantification of AZM198 in whole blood, drawn at 8 a.m. and 5 p.m. after 3 days of feeding in a pilot study and after 21 days of feeding in the main study (data not shown).

### 2.12. Statistics

Results are expressed as mean ± SEM. Statistical analysis was performed using one way ANOVA (when assuming Gaussian distribution) followed by appropriate post hoc tests. All calculations were performed using GraphPad Prism version 8.4. * *p* < 0.05, ** *p* < 0.01, *** *p* < 0.001, **** *p* < 0.0001. Histological and echocardiographic data was evaluated by two independent individuals in a blinded fashion.

## 3. Results

### 3.1. Inflammatory Response and Cardiac Neutrophil Infiltration

**Figure 1:**
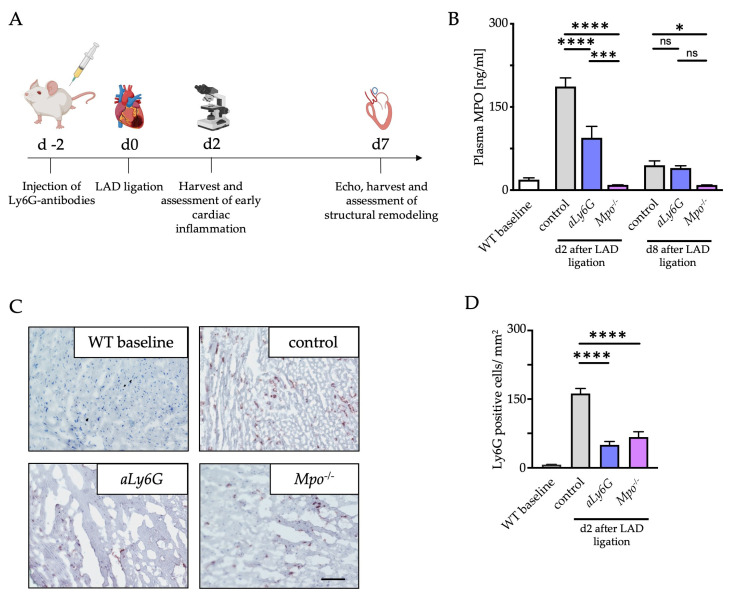
Polymorphonuclear neutrophil (PMN) reduction and myeloperoxidase (MPO) deficiency (*Mpo*^−/−^) alleviate inflammatory response after myocardial infarction (MI). (**A**) Schematic timeline of Ly6G antibody treatment, permanent left anterior descending artery (LAD) ligation (PI) and cardiac analyses: wildtype (WT) mice were treated with Ly6G antibodies 2 days before PI operation followed by cardiac investigations at days 2 and 7. (**B**) MPO plasma levels 2 and 8 days after PI assessed by ELISA. (**C**) Representative immunohistochemical Ly6G stainings (brown) of cardiac sections after 2 days of PI. Scale bar = 200 µm. (**D**) Quantitative analysis of Ly6G positive cells within the infarct and peri-infarct region 2 days after PI. n = 5/5/5/5; * *p* < 0.05, *** *p* < 0.001, **** *p* < 0.0001, ns = not significant; one way ANOVA test followed by appropriate post hoc test, mean ± SEM is shown.

To characterize the influence of PMN reduction on cardiac performance and remodeling after MI, mice were treated with one Ly6G antibody injection 2 days before permanent ligation (PI) of the LAD (Figure 1A). Antibody treatment resulted in an appr. 85% reduction of PMN for up to 8 days (Supplemental Figure S1). To clarify the role of MPO, an important mediator of inflammation and most abundantly expressed in PMN [13], *Mpo*^−/−^ mice were used accordingly. MI is a potent inducer of inflammation [12,29]. To evaluate the inflammatory response, MPO plasma levels were measured 2 and 8 days after LAD ligation in WT control (control), WT Ly6G antibody treated (*aLy6G*) and *Mpo*^−/−^ mice. Plasma levels were significantly increased 2 days after LAD ligation and approached baseline levels after 8 days. Of note, Ly6G antibody injection significantly reduced MPO plasma levels 2 days after LAD ligation (Figure 1B). To investigate the effect of Ly6G injection on PMN infiltration in murine hearts after PI, staining of Ly6G positive cells in the infarct and peri-infarct zones was performed, indicating a markedly attenuated infiltration of PMN in *aLy6G* and *Mpo*^−/−^ mice compared to control animals up to 2 days after PI (Figure 1C,D).

### 3.2. Influence of Neutrophils and MPO on Cardiac Function

**Figure 2:**
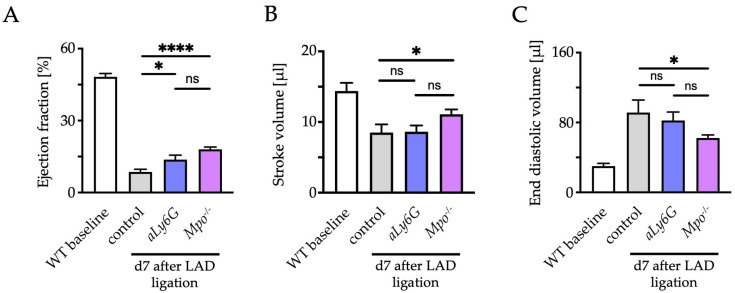
Echocardiographic assessment of (**A**) left ventricular (LV) ejection fraction, (**B**) LV stroke volume and (**C**) LV end diastolic volume 7 days after PI. n = 4/10/8/11; * *p* < 0.05, **** *p* < 0.0001, ns = not significant; one way ANOVA test followed by appropriate post hoc test, mean ± SEM is shown.

To assess the effect of PMN reduction on heart function, echocardiography was performed at baseline and 7 days after LAD ligation in control, *aLy6G* and *Mpo*^−/−^ mice. Left ventricular ejection fraction (LV-EF) showed a substantial decline after MI in all groups but was significantly less impaired in *aLy6G* mice as compared to control animals with a similar effect to that of *Mpo*^−/−^ animals (Figure 2A). Accordingly, stroke volume was significantly higher and end diastolic volume significantly smaller in *Mpo*^−/−^ animals compared to controls, whereas no difference was detectable between *aLy6G* and WT control hearts (Figure 2B,C). Heart rate, body weight or ventricle length were similar in all groups after LAD ligation (Supplemental Figure S2A–C).

### 3.3. Left Ventricular Fibrosis

**Figure 3:**
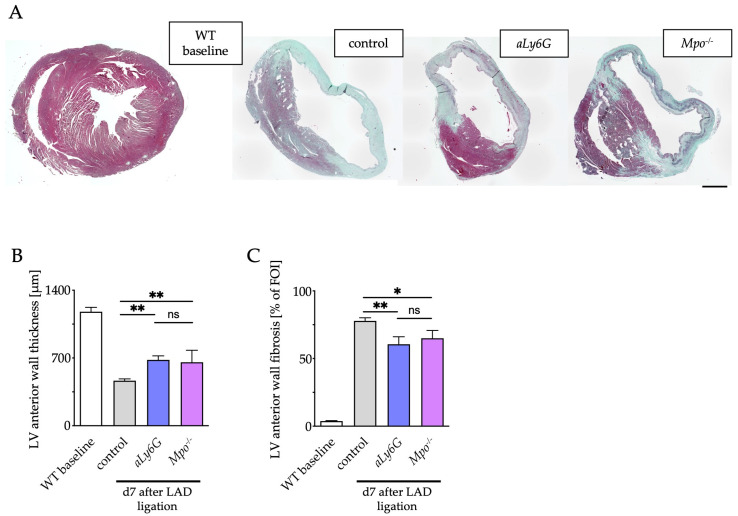
Structural remodeling after MI. (**A**) Representative Masson’s trichrome staining of murine hearts of WT baseline and after 7 days of PI. Scale bar: 1 mm. (**B**) Quantification of LV anterior wall thickness and (**C**) of left ventricular fibrotic areas. n = 4/8/8/8; * *p* < 0.05, ** *p* < 0.01, ns = not significant; one way ANOVA test followed by appropriate post hoc test, mean ± SEM is shown.

Post ischemic fibrotic remodeling is a major cause for deterioration of heart function [30]. Therefore, fibrosis was evaluated by trichrome staining at baseline and 7 days after LAD ligation. PMN reduction resulted in less atrophy of the myocardium, as evidenced by a significantly smaller decrease in LV wall thickness similar to that of *Mpo*^−/−^ animals as compared to control hearts (Figure 3A,B). Furthermore, LV fibrosis was more pronounced in control hearts as compared to *aLy6G* and *Mpo*^−/−^ mice (Figure 3C).

### 3.4. Myofibroblast Accumulation

**Figure 4:**
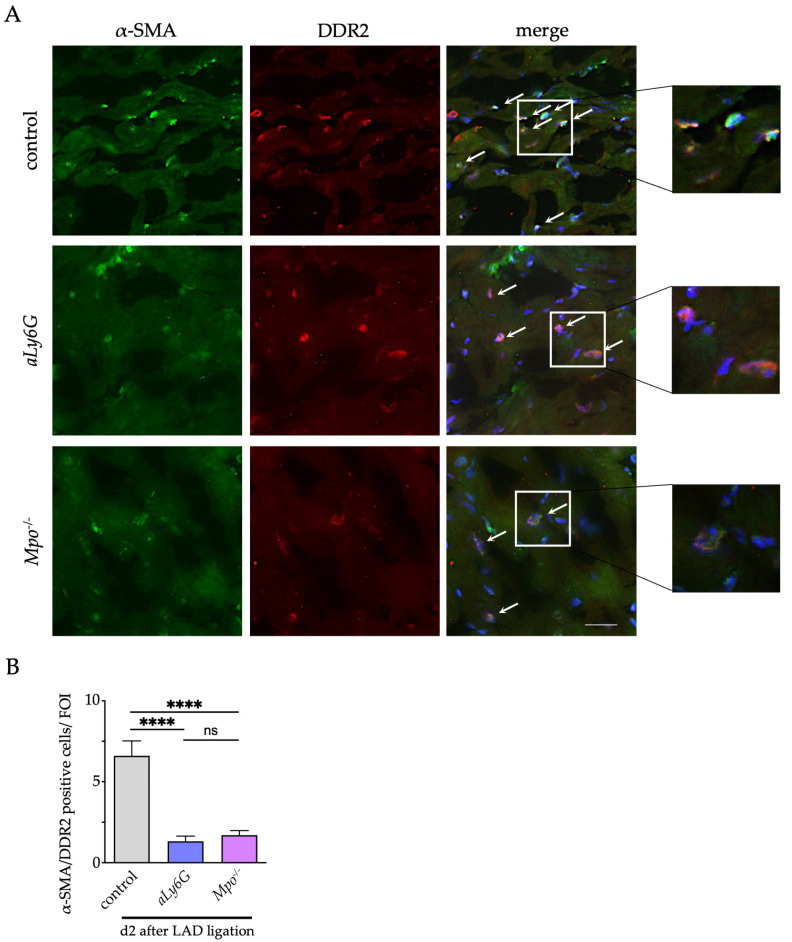
Myofibroblast transdifferentiation after MI. (**A**) Representative immunofluorescence stainings of murine cardiac sections 2 days after PI for the fibroblast marker discoidin domain-containing receptor 2 (DDR-2; green) and the myofibroblast marker α–smooth muscle actin (α-SMA; red) (blue = DAPI; scale bar = 100 μm). Arrows indicate double positive cells. (**B**) Quantification of myofibroblasts within the infarct and peri-infarct region. n = 5/5/5; **** *p* < 0.0001, ns = not significant; one way ANOVA test followed by appropriate post hoc test, mean ± SEM is shown.

Tissue replacement and interstitial fibrosis after myocardial injury is mainly driven by activation of cardiac fibroblasts following transdifferentiation to myofibroblasts, a process driven by PMN and MPO [18,31]. To determine the number of cardiac myofibroblasts, co-localization of the fibroblast marker discoidin domain-containing receptor (DDR-2) and the myofibroblast marker α-smooth muscle actin (α-SMA) were assessed in cardiac sections 2 days after LAD ligation. Consistent with the observed lower extent of fibrosis, the myofibroblast count was significantly lower in the infarct and peri-infarct region of *aLy6G* and *Mpo*^−/−^ than in control myocardium. Of note, no difference between *aLy6G* and *Mpo*^−/−^ animals could be detected (Figure 4A,B).

### 3.5. Connexin 43 in the Peri-Infarct Area

**Figure 5:**
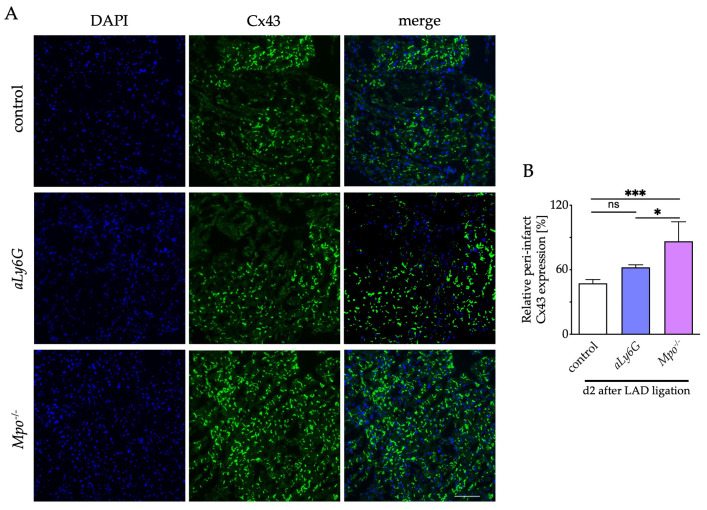
Connexin 43 (Cx43) expression in peri-infarct region. (**A**) Representative immunofluorescence stainings of murine cardiac sections after 2 days of PI for Cx43 (green = Cx43, blue = DAPI; scale bar = 50 μm). (**B**) Quantitative analysis of relative Cx43 expression within the peri-infarct region compared with healthy myocardium. n = 6/4/5; * *p* < 0.05, *** *p* < 0.001, ns = not significant; one way ANOVA test followed by appropriate post hoc test, mean ± SEM is shown.

Connexins are an essential component of cell–cell communication and conduction homogeneity in myocardial tissue, and a reduced expression has been associated with the development of arrhythmic events after myocardial injury [18,32]. To investigate the effect of PMN reduction on electrical cardiac remodeling, we measured the expression of connexin 43 (Cx43) within the conduction relevant peri-infarct region in relation to Cx43 content in healthy myocardial tissue 2 days after LAD ligation. Interestingly, MPO deficiency significantly protected from Cx43 degradation after LAD-ligation, whereas PMN reduction only numerically increased Cx43 levels as compared to control hearts (Figure 5).

### 3.6. Pharmacological MPO Inhibition

**Figure 6:**
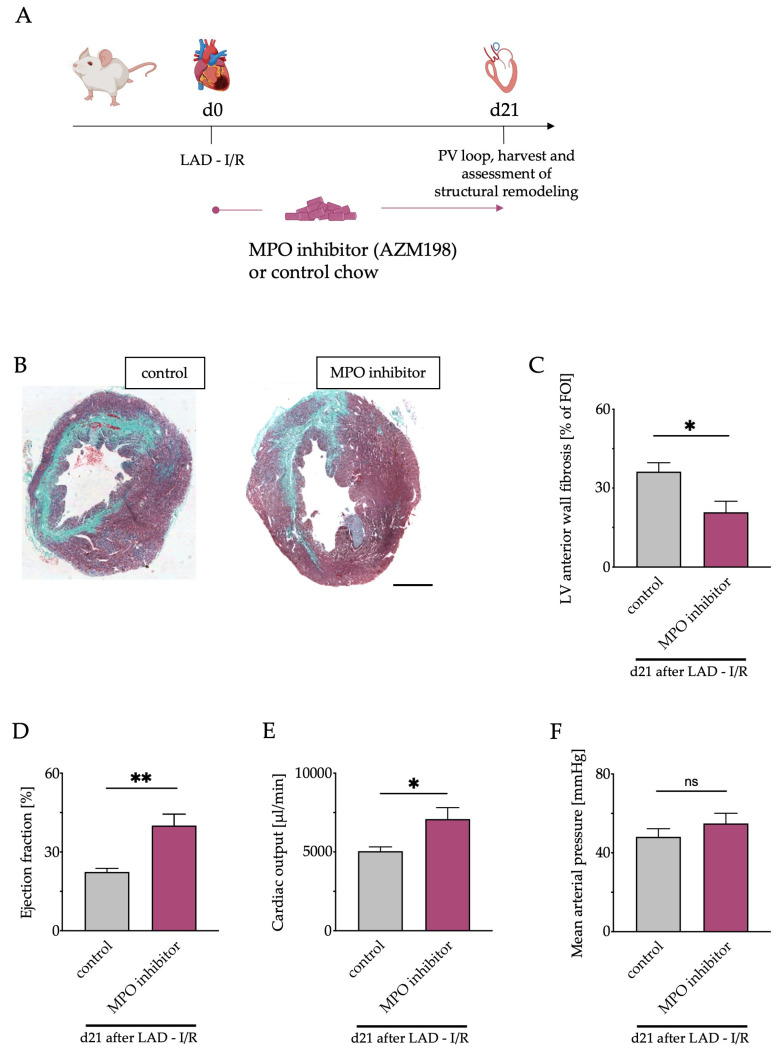
Pharmacological MPO inhibition mitigates cardiac remodeling and functional deterioration after MI. (**A**) Schematic timeline of MPO inhibitor treatment (AZM198) followed by LAD ligation and reperfusion (I/R) and final cardiac investigations. (**B**) Representative Masson’s trichrome stainings of murine hearts 21 days after I/R; scale bar = 1 mm. (**C**) Quantification of left ventricular fibrotic areas. (**D**) Quantitative analysis of LV ejection fraction, (**E**) cardiac output, (**F**) mean arterial pressure 21 days after I/R assessed by pressure–volume (PV) loop analysis. n = 5/7; * *p* < 0.05, ** *p* < 0.01, ns = not significant; one way ANOVA test followed by appropriate post hoc test, mean ± SEM is shown.

Given the increasing number of interventional coronary reperfusion therapies in the context of acute MI, we aimed to apply our findings to a more translational model of transient ischemia. Hence, mice were subjected to LAD ligation for 40 min followed by reperfusion (I/R). Furthermore, we aimed to translate our findings into a therapeutic approach. Since results of *Mpo*^−/−^ and *aLy6G* mice were comparable and the clinical application of MPO inhibitors is within reach, being currently investigated in phase II clinical trials, WT mice were fed an MPO inhibitor (AZM198) or control chow for 21 days after I/R (Figure 6A). In this way, relevant changes in the late stages after MI can be evaluated, which are largely influenced by MPO, as suggested by recent studies [11].

### 3.7. Left Ventricular Fibrosis and Cardiac Function after Pharmacological MPO Inhibition

Trichrome staining of murine hearts 21 days after I/R showed significantly less LV fibrosis in mice fed AZM198 compared to control diet (Figure 6B,C). To evaluate the impact of the observed structural differences between the two groups on cardiovascular function and hemodynamic parameters, pressure–volume loop analyses were performed. LV ejection fraction and cardiac output were significantly higher in AZM198-treated mice (Figure 6D,E). No difference in mean arterial pressure (MAP) was observed (Figure 6F).

## 4. Discussion

In this study, we investigated the influence of PMN reduction, MPO deficiency and therapeutic MPO inhibition on cardiac integrity after MI in a model of PI and I/R. Cardiac myocyte cell death is a potent activator of an innate immune response. Subsequent infiltration of the ischemic area with PMN, monocytes and macrophages, dendritic cells and lymphocytes promotes the initiation and resolution of inflammation, removal of debris, angiogenesis and ventricular remodeling, which in the end determines the degree of cardiac functional deterioration [30,33]. The role of PMN and their inflammatory and regulatory capacities in this context ranges between beneficial and deleterious effects, and their delicate interplay is not well understood. Because of their ability to induce oxidative stress in tissues, mainly destructive effects on the myocardium have long been suspected [8]. Consistent with this notion, beneficial effects on cardiac function have been observed after modulation of their inflammatory activity by inhibiting oxidative enzymes [11]. In contrast, complete depletion of PMN has surprisingly resulted in an increased scar formation and poorer left ventricular (LV) function after infarction due to an inhibited reparative capacity [10].

Here, we examine the effects of PMN reduction rather than complete depletion. For this purpose, in contrast to repetitive Ly6G antibody administration in previous studies [10], only a single antibody treatment 2 days before LAD ligation was performed, which lead to a sufficient reduction in numbers but not to a complete loss of PMN. Accordingly, decreased plasma MPO levels were measured. Here, investigation of the inflammatory response in the ischemic myocardium showed less PMN infiltration after treatment with Ly6G antibodies. In addition, less fibrotic remodeling was observed. Further elucidation of the underlying mechanisms revealed a decreased number of cardiac myofibroblast. PMN can activate p38 mitogen-activated protein kinase (MAPK) in a HOCl-dependent manner via MPO [34], which promotes transdifferentiation of fibroblasts to myofibroblasts [18], key cellular drivers of interstitial collagen production and fibrosis after myocardial ischemia [31]. The extent of maladaptive structural remodeling is critically linked to myocardial functionality [30]. Accordingly, we found improved LV systolic function after PMN reduction. In summary, we could demonstrate that reduction, but not complete depletion, of PMN improves structural remodeling and heart function after PI.

Post-infarction malignant ventricular arrhythmias are an important cause of mortality in western countries [35]. Connexons, consisting of six single connexins, are ion channels that are essential for the myocardial conduction system [36]. Preserved expression of Cx43 is associated with reduced susceptibility to ventricular tachycardia [18,37]. Previous data show that Cx43 is degraded by matrix metalloproteinases, which can be activated by MPO-derived HOCl [38]. Interestingly, no significant difference in Cx43 stability was detectable between Ly6G antibody-treated (*aLy6G*) and untreated mice after MI, whereas preserved Cx43 expression was seen in *Mpo*^−/−^ mice, indicating a potential sensitivity of Cx43 to residual MPO after PMN reduction. This may have implications for therapeutic treatment approaches with Ly6G antibodies after MI and needs to be monitored in future studies.

MPO is a crucial mediator of the inflammatory capacities of PMN [14]. In *Mpo*^−/−^ mice, comparable protective effects were seen with respect to PMN infiltration, fibroblast-to-myofibroblast transdifferentiation and LV fibrosis after PI. Functionally, LV systolic function improved to a similar level as in *aLy6G* animals, and higher LV stroke volume and lower LV dilation were observed in *Mpo*^−/−^ animals as compared to control and *aLy6G* animals. Additionally, enhanced Cx43 stability could be found in *Mpo*^−/−^ mice but not in *aLy6G* animals. Taking into account the residual MPO plasma levels in *aLy6G* animals after PI, these results might indicate that the protective cardiac effect of PMN reduction can be explained to some extent by reduced MPO activity. This is supported by earlier studies indicating a pathophysiological role of MPO in the context of atrial fibrillation, HF, responsiveness to resynchronization therapy and risk prediction in acute coronary syndromes [39,40,41,42].

To extend these findings to a potential therapeutic level, we studied MPO inhibition in a clinically more relevant model of myocardial I/R injury due to the increasing availability of interventional reperfusion therapies. MPO inhibitors are subject to current preclinical and clinical studies, and protective effects of a particular MPO inhibitor on postinfarction cardiac function have already been demonstrated in a murine model of PI [11,21,43]. Herein, we are the first to test the thioxanthine derivative AZM198, an orally administered MPO inhibitor, in the context of I/R. The crucial question we addressed in this context was whether therapeutic MPO inhibition could reduce the increasing incidence of ischemic cardiomyopathy and HF after MI due to their enormous impact on the healthcare system in western countries [3,4]. Therefore, we examined the effect of an MPO-inhibitor treatment beyond the acute phase of up to 21 days after I/R. For functional assessment, we performed pressure–volume analyses to also obtain hemodynamic parameters, which allows MPO’s important effects on vascular function to also be considered [44]. Interestingly, a marked preservation of LV systolic function and cardiac output was detectable after MPO inhibitor treatment. On a structural level, a significant reduction in LV fibrosis could be observed compared with the control group 21 days after I/R, which could mechanistically be explained by decreased MPO-mediated p38 MAPK activation and myofibroblast transdifferentiation, consistent with the results of PMN reduction [34].

Previous data show that vascular tone is critically mediated by the interplay of MPO activity and NO signaling [44]. NO is an important vasodilator and its bioavailability is limited by MPO [13,45]. Therefore, MPO inhibition could lead to vasodilation via attenuated NO consumption with subsequent reduction in cardiac afterload and arterial pressure [46]. A potential decrease in MAP is of clinical relevance since a major target of classical HF medication is lowering of an inadequately elevated neurohumoral activation, thereby also attenuating blood pressure [47]. The reduction in blood pressure in particular is a frequent limiting factor that restricts the continuation or escalation of HF therapy [48]. Therefore, special attention was paid to the influence of MPO inhibition on MAP. Remarkably, no difference in the MPO inhibitor-treated and control groups was detectable, suggesting that any potential reduction in vascular tone may have been offset by improved cardiac output, thereby maintaining a stable blood pressure level.

### 4.1. Conclusions

Our results demonstrate that antibody-induced reduction of PMN numbers shows protective cardiac effects after MI. MPO, as one of their most abundantly expressed proteins [49], might play a crucial role in this context. Therapeutic MPO inhibition was able to protect cardiac integrity after I/R and resulted in significant improvement of LV systolic function and cardiac output.

Among the underlying mechanisms, attenuated myocardial scar formation via reduced fibroblast-to-myofibroblast transdifferentiation appears to be of relevance. Strikingly, MPO inhibition had no lowering effect on arterial blood pressure in this model, which in many cases limits the therapeutic options of classical HF therapy.

Consequently, in the setting of MI, MPO emerges as a favorable pharmacological target for the prevention of long-term morbidity. Further therapeutic evaluation seems promising based on the current data.

### 4.2. Limitations

The Ly6G antibody treatment in here was performed 2 days before MI and was therefore not investigated in a therapeutic approach. Future studies could include different time points and dosages of antibody administration to further elucidate the therapeutic potential of a PMN reduction in the context of MI. Therapeutic MPO inhibition has come more and more into scientific focus. The data presented here based on the orally administered inhibitor AZM198 show promising results regarding a protective effect in myocardial ischemia in a murine model of I/R. Nevertheless, murine models can only partly resemble the human physiology. The immediate time point after I/R was chosen as the onset of therapy. In clinical reality, this is not always possible in many cases, for example, when MI has proceeded quietly. The optimal time of initiation of therapy currently remains unknown and should be evaluated in future studies. Therapeutic evaluation in a chronic model of ischemic cardiomyopathy, in which therapy is initiated only after the onset of HF, would also be of interest.

## Data Availability

The data are contained within the manuscript and supplementary files.

## Supplementary Materials

The following supporting information can be downloaded at: https://www.mdpi.com/article/10.3390/antiox12010033/s1, Figure S1: Neutrophil count after Ly6G antibody treatment; Figure S2: Further physiological and phenotypic evaluation after PI.
