# Supplementary material for: Protective Effects of Therapeutic Neutrophil Depletion and Myeloperoxidase Inhibition on Left Ventricular Function and Remodeling in Myocardial Infarction
Source: Antioxidants (Basel). 2022 Dec 24;12(1):33. doi: 10.3390/antiox12010033 (PMC9854671; doi:10.3390/antiox12010033)
Supplement: Supplementary file 1 [file antioxidants-12-00033-s001.zip › antioxidants-2075189-supplementary.pdf]

Supplement

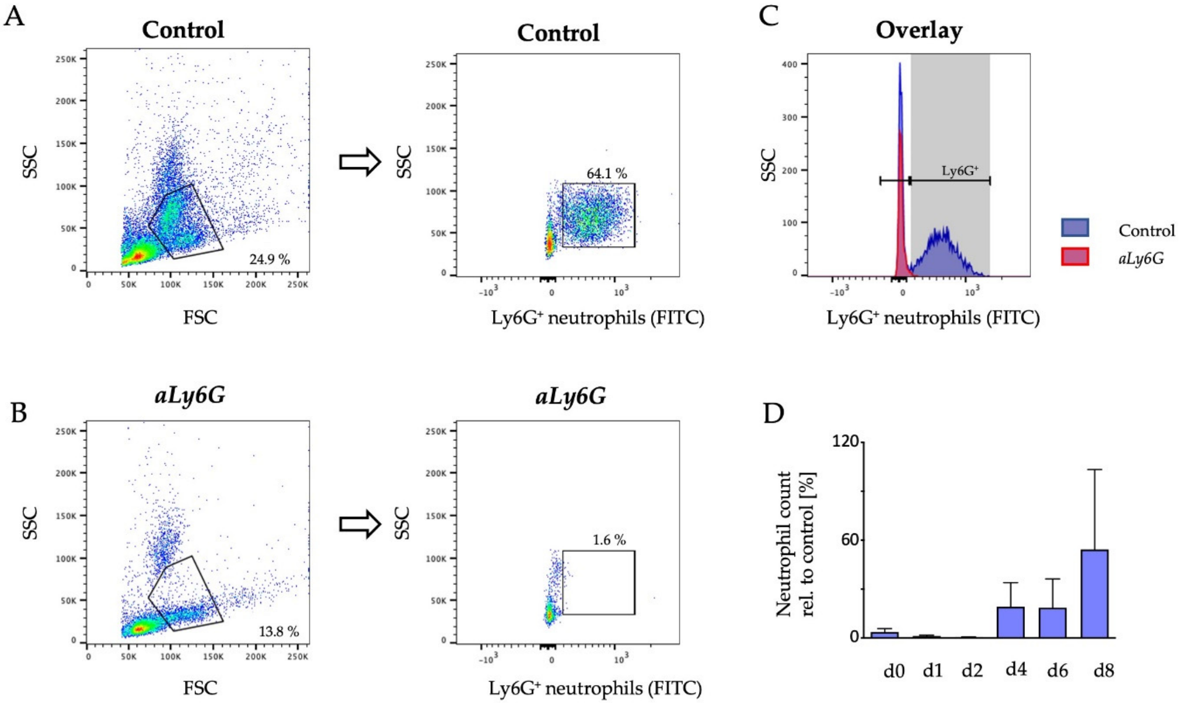

Supplemental Figure S1. Neutrophil count after Ly6G antibody (*aLy6G*) treatment. WT Mice were treated with Ly6G-antibodies via intraperitoneal injection and neutrophil count was measured by FACS analysis. Neutrophil populations in control (A) and *aLy6G* treated mice (B) were identified by FITC staining of classical PMN marker Ly6G. Representative overlay of Ly6G$^+$ neutrophil populations at day 2 revealed significant differences in neutrophil populations (C). Neutrophil counts relative to control mice on the indicated days showed a temporary suppression of Ly6G$^+$ after antibody treatment (D). n = 4/3/8/4/3/3; mean ± SEM is shown.

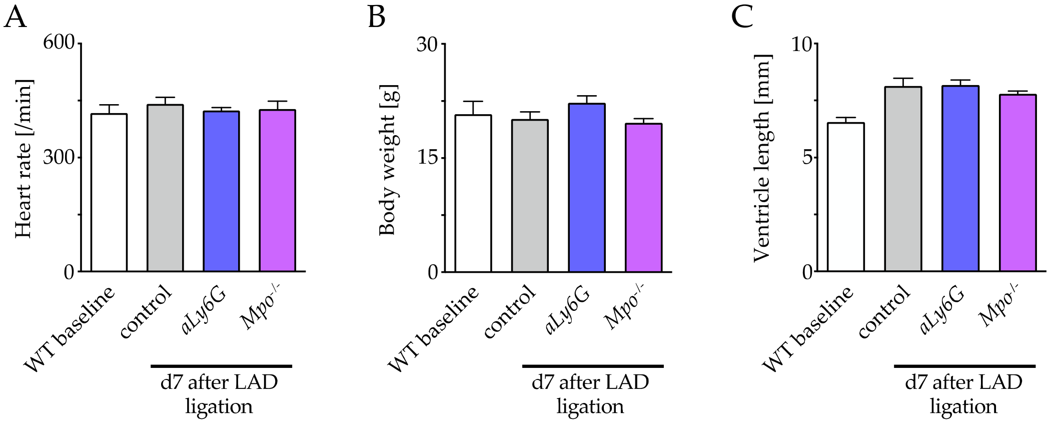

Supplemental Figure S2. Further physiological and phenotypic evaluation after PI. Quantitative analyses of (A) heart rate, (B) body weight and (C) ventricle length in the indicated groups. n = 6/8/8/7; mean ± SEM is shown.
